# The impact of dolutegravir on the growth of HIV-exposed uninfected infants: an observational cohort study in rural Tanzania

**DOI:** 10.1016/j.eclinm.2026.103984

**Published:** 2026-05-28

**Authors:** Sara Glanzmann, Ezekiel Luoga, James Okuma, Elizabeth Dotto, George Sigalla, Fabian Christoph Franzeck, Emanuel Nyenza, Dorcas Mnzava, Mathias Bukuku, Lilian Moshi, Lulu Wilson, Daniel H. Paris, Tracy R. Glass, Getrud Joseph Mollel, Maja Weisser, Fiona Vanobberghen, KIULARCO Study Group, Aschola Asantiel, Farida Bani, Manuel Battegay, Mathias E. Bukuku, Theonestina Byakuzana, Adolphina Chale, Joyce Claud, Elizabeth K. Dotto, Kabula A. Elias, Gideon Francis, Tracy Glass, Yvonne Haridas, Speciosa Hwaya, Gift Joseph, Rodney M. Julius, Fatuma Kabelele, Aneth V. Kalinjuma, Andrew Katende, Fredrick Kazetera, Yassin Kisunga, Bernard Kivuma, Thomas Klimkait, Juma Kupewa, Ezekiel Luoga, Jerome Lwali, Clarence A. Mahundo, Mkiwa A. Makwea, Edgar E. Martin, Honorati Masanja, Swalehe Masoud, Efrazia Mayanzani, Mohammed Mbaruku, Geofrey Mbunda, George P. Mfanando, Josephine Mhina, Mengi Mkulila, Margareth Mkusa, Christina Mluge, Franzisca A. Mmbando, Alpha Mninje, Lina Mnunga, Dorcas K. Mnzana, Getrud J. Mollel, Lilian Moshi, Germana Mossad, Regina Mponji, Dolores Mpundunga, Athumani Mtandanguo, Ummu-kulthum Mwaliga, Alice I. Mwanga, Sanula Nahota, Sharifa Nakapala, Regina Ndaki, Robert C. Ndege, Happyphania Ngakongwa, Agatha Ngulukila, Alex John Ntamatungiro, Emmanuel Nyenza, James Okuma, Ally Olotu, Daniel H. Paris, Albert Raphael, Martin Rohacek, Leila Samson, Elizabeth Senkoro, George Sigalla, Jamali B. Siru, Jenifa Tarimo, Juerg Utzinger, Fiona Vanobberghen, Maja Weisser, John Wigay, Lulu Wilson

**Affiliations:** a Division of Infectious Diseases, University Hospital Basel, Basel, Switzerland; b Ifakara Health Institute, Ifakara, Tanzania; c St. Francis Regional Referral Hospital, Ifakara, Tanzania; d Swiss Tropical and Public Health Institute, Allschwil, Switzerland; e University of Basel, Basel, Switzerland; f Research and Analytics, Department of Informatics, University Hospital Basel, Basel, Switzerland; g Department of Infectious Diseases, University Medical Center Basel-Land, Liestal, Switzerland; h Charité – University Medicine Berlin, Berlin, Germany; i St Francis University College of Health and Allied Sciences, Ifakara, Tanzania

**Keywords:** HIV-exposed uninfected children, Growth, Dolutegravir, Efavirenz, Pregnancy

## Abstract

**Background:**

Antiretroviral treatment (ART) in pregnant and breastfeeding women living with HIV exposes their HIV-exposed uninfected (HEU) infants to drug, which might affect their outcomes. We evaluated the effects of maternal dolutegravir-based versus efavirenz-based ART and associated factors on growth outcomes in HEU children born to women living with HIV in rural Tanzania.

**Methods:**

In this observational cohort study, we used data from the prospective Kilombero and Ulanga Antiretroviral Cohort (KIULARCO) to assess growth outcomes (up to age 18 months) of HEU children born to women living with HIV attending the Chronic Diseases Clinic of Ifakara in rural Tanzania between May 2012 and December 2024. Recommended first-line ART changed from efavirenz-based to dolutegravir-based from 2019 onwards. We included follow-up through database extraction on May 3, 2025. The primary outcomes were length-for-age and weight-for-length z-scores, defined using WHO child growth standards, and secondary outcomes included stunting and wasting, defined as >2 standard deviations below the WHO standard. We modelled continuous outcomes using linear random effects models and binary outcomes using logistic generalised estimating equations.

**Findings:**

Among 1019 infants born to 844 mothers, 491 (48%) mother–infant pairs were included in the efavirenz group and 528 (52%) in the dolutegravir group. Maternal ART was initiated before pregnancy in 569 (56%) mother–infant pairs, during pregnancy in 339 (33%), and during labour or after delivery in 111 births (11%). At delivery, median maternal age was 31 years (interquartile range 26–36) and 742 (78%) of mothers had a WHO stage I/II infection. Overall, 526 (52%) infants were male and 968 (96%) born at term. Infants had lower length and weight trajectories compared to WHO reference standards, irrespective of gender or ART regimen. In descriptive analyses, the prevalence of stunting measured at each visit over the first 18 months of life ranged between 18 and 31% in the efavirenz group and 8–26% in the dolutegravir group, possibly confounded by enrolment in later calendar years in the dolutegravir group. In adjusted models, we observed no difference in growth outcomes by ART regimen.

**Interpretation:**

In this large cohort-based study, we found no association between maternal ART regimen and growth of HEU infants, but growth remained below WHO reference standards. Our findings support the continued use of dolutegravir as the preferred first-line ART and emphasise the need for further research and targeted interventions to improve the health of the growing population of HIV-exposed uninfected infants.

**Funding:**

The Kilombero and Ulanga Antiretroviral Cohort has received funding from the Canton Basel-Stadt, the Swiss Tropical and Public Health Institute and the University Hospital Basel. This study was funded by the 10.13039/501100016071Goldschmidt-Jacobson Foundation and by the Mathieu Foundation. The Foundations were not involved in the study design, data collection, data analysis or writing of the manuscript. No other funding has been received.

**Research in context**

**Evidence before this study**

To identify the impact of dolutegravir or efavirenz on birthweight and weight gain in HIV-exposed non-infected infants, we did a PubMed search on 8 June 2025 using the following terms (“child∗” OR “infant∗”) AND (“dolutegravir” [Supplementary Concept] OR Dolutegravir[tw] OR “integrase inhibitor” [tw]) AND (Efavirenz[tw] OR “non-nucleoside reverse transcriptase inhibito∗” [tw] OR NNRTI∗[tw]) AND (“Birth weight” [tw] OR stunting[tw] OR height[tw] OR “Infant, Low Birth weight”[Mesh] OR “weight” [tw]). Out of 25 articles identified, we excluded 15 studies which analysed adults or maternal outcomes, three studies performed in HIV-positive children, and two studies on adverse birth outcomes not specifying weight/growth aspects. Within the randomised controlled DolPHIN-1 trial evaluating safety and pharmacokinetics of dolutegravir versus efavirenz initiated in the third trimester among 60 pregnant women with HIV, authors found comparable birthweights of 3 kg (IQR 2–4) of their neonates. As a secondary analysis of the randomised-controlled IMPAACT 2010 (VESTED) trial, researchers assessed growth at 26 and 50 weeks in 577 HIV-exposed uninfected children. Length-for-age and weight-for-age z-scores were significantly higher (mean difference 0.3–0.4 points) through week 50 in infants from mothers randomised to a dolutegravir- versus efavirenz-containing regimen, while weight-for-length was similar in both groups. A cohort of mother infant-pairs from Kenya found lower growth parameters in the 1000 HIV-exposed uninfected children versus 100 HIV non-exposed children within the first year, but no impact of the maternal antiretroviral drug regimen used (dolutegravir-, efavirenz- and protease inhibitor/other-based) on growth in HIV-exposed infants. In a cohort of 292 pregnant women from South Africa, there was no evidence of a difference in infant birthweight between women on a dolutegravir- versus efavirenz-based regimen, although higher maternal gestational weight gain was observed in the dolutegravir study arm. A pharmacovigilance study including 34 case reports had too few pregnant women on integrase inhibitors to analyse potential differences in birthweight. Further literature review also yielded the DolPHIN-2 trial, which evaluated virological outcomes between mothers randomised to dolutegravir- versus efavirenz-based regimens initiated in the third trimester, and found similar birthweights between the groups of around 3.1–3.2 kg.

**Added value of this study**

In our study of 1019 HIV-uninfected children born to women living with HIV on dolutegravir- or efavirenz-based antiretroviral regimens, we found no evidence of a difference in length-for-age or weight-for-length z-scores through 18 months of follow-up. Our study adds to the existing literature with a long follow-up of infant growth parameters from birth, in a high proportion of women who started ART at the pre-conception stage and in a rural setting, where underweight is more prevalent than in urban settings.

**Implications of all the available evidence**

HIV-exposed but uninfected infants have poorer growth outcomes compared to children not exposed to HIV. Dolutegravir is known to cause weight gain in adults but appears to have limited impact on birthweight and growth in infants from mothers on dolutegravir-based regimens during pregnancy and breastfeeding compared to regimens not containing dolutegravir. Our findings support the continued use of dolutegravir as the preferred first-line ART and emphasise the need for further research and targeted interventions to improve the health of the growing population of HIV-exposed uninfected infants.

## Introduction

The prevention of vertical transmission of HIV is an outstanding success in the fight against HIV.^1^ The introduction of antiretroviral treatment (ART) for pregnant women living with HIV, and prophylactic ART in the first six weeks of life for the HIV-exposed infant, has reduced the risk of vertical HIV transmission to <15% in most middle-income and low-income countries,^2^ holding promise to eliminate vertical HIV transmission. Consequently, the population of HIV-exposed uninfected (HEU) children is increasing. Today, HEU infants greatly outnumber those living with HIV, with an estimated 16.1 million HEU infants worldwide compared to 2.4 million children living with HIV.^3^^,^^4^

Despite being uninfected, HEU children have poorer health outcomes compared to HIV-unexposed uninfected children, including higher mortality rates, higher infectious morbidity, greater neurodevelopment impairment and poorer growth outcomes such as lower birthweight and higher risk of stunting and wasting.^3^^,^^5^, ^6^, ^7^, ^8^, ^9^, ^10^ Factors contributing to these outcomes are multifactorial presumably including maternal immune dysregulation, exposure to maternal ART, and socioeconomic challenges such as nutritional factors.^8^^,^^9^^,^^11^ A study from Botswana found lower height-for-age and weight-for-age z-scores in HEU infants aged 24 months who were exposed to maternal combination ART (non-nucleoside reverse transcriptase inhibitor or protease inhibitor-based ART) compared to those exposed to zidovudine monotherapy.^12^ While maternal viral suppression through ART for prevention of vertical HIV transmission remains key, such a finding prompts concerns about the long-term health implications for HEU infants exposed to ART in-utero and during breastfeeding and the question arises whether the choice of maternal ART affects HEU infant outcomes.^8^^,^^9^

In adults, especially females including pregnant women, dolutegravir (DTG)-based regimens are associated with higher weight gain compared to efavirenz (EFV)-based regimens.^13^, ^14^, ^15^ Whether the use of DTG in pregnant and breastfeeding women living with HIV affects the growth outcomes of their HEU children has not yet been well studied, and most studies report on birthweight or short time periods after birth only.^16^, ^17^, ^18^, ^19^ Weight gain in infants could be beneficial in settings with high rates of undernutrition. We aimed to evaluate the effect of maternal DTG-based versus EFV-based ART and associated factors on growth outcomes in HEU children born to women living with HIV in rural Tanzania.

## Methods

### Study design and setting

In this observational cohort study, we analysed mother–infant pairs enrolled in the prospective Kilombero and Ulanga Antiretroviral Cohort (KIULARCO), a cohort of people living with HIV (PLHIV) seen at the Chronic Disease Clinic of Ifakara (CDCI) at the St. Francis Regional Referral Hospital in Ifakara, Tanzania.^20^^,^^21^ Infants born to women enrolled in KIULARCO are followed as part of the cohort until 2 years of age. Final HIV testing is done at 18–24 months of age as per World Health Organization (WHO) and Tanzanian national guidelines.^22^^,^^23^

### Ethics

Informed consent was obtained from all mothers at enrolment into KIULARCO to approve the use of their data plus the data from their infant within the first 2 years of life. Ethical approval for KIULARCO is granted annually by the local Institutional Review Board (IHI/IRB/No16-2006) and the National Institute of Medical Research (NIMR/HQ/R.8a/Vol.IX/620).

### Participants

We included all mother–infant pairs with HEU infants registered from May 2012 through to December 2024, whose mothers consented for enrolment into KIULARCO and initiated DTG- or EFV-based ART before the infant reached six months of age. Mother-infant pairs were excluded if the infant was diagnosed with HIV by DNA PCR or antibody test at any point during follow-up. We included follow-up through database extraction on May 3, 2025.

### Study procedures

Within routine care, pregnant and breastfeeding women with their infant are scheduled for monthly visits until 18 months of infant life, starting with infant registration at 6 weeks of age. Birthweight is recorded by the labour ward nurse on the antenatal care record which the mother brings to postnatal care and is then captured in the electronic medical record system (openMRS). Sociodemographic, clinical, and laboratory data are captured prospectively in openMRS. At every visit, infant length and weight are recorded in a standardised measurement following WHO guidelines.^24^ Additionally, infants receive vaccinations, ART and cotrimoxazole prophylaxis, and HIV testing; and mothers receive infant feeding practice recommendations and viral load (VL) testing (see Supplementary Methods).

### Outcomes and exposure variables

The primary outcomes were length-for-age and weight-for-length z-scores, defined using WHO child growth standards.^25^^,^^26^

Secondary outcomes were the prevalence of moderate and severe stunting and wasting, defined as >2 and >3 standard deviations below the WHO length-for-age and weight-for-length child growth standards, respectively^27^^,^^28^; and birthweight. The exposure of interest was the maternal ART regimen, hereafter indicated as “DTG” or “EFV” groups, based on the regimen at the time of delivery. Prior to 2019, the recommended first-line ART was EFV-based, thereafter changing to DTG-based, initially upon the mother’s consent due to concerns surrounding a potential link between DTG and neural tube defects, and later DTG-based for all once these concerns were refuted.^29^, ^30^, ^31^ Completion of follow-up at 18 months was defined as having a final infant HIV test and/or a vitals measurement at that timepoint.

### Statistical analysis

We summarised mothers’ characteristics at delivery and enrolment into KIULARCO (see Supplementary Methods), infant characteristics at birth, maternal ART changes, mothers’ VL during the breastfeeding period, infant feeding, and infant outcomes. We illustrated infant growth graphically. For descriptive purposes, z-score data were categorised into three-monthly intervals (window ± 1.5 months, and up to 30 months for the final 18-month visit); for modelling, all z-score data up to 30 months were used. Adjusted linear random effects models were used to model the repeated continuous z-score outcomes and logistic generalised estimating equations for the repeated binary outcomes of stunting and wasting. We used these models to predict marginal mean z-scores or the probability of stunting or wasting, for DTG and EFV groups, adjusted for confounders. Models were adjusted for variables considered to be confounders for the relationship between ART group and outcomes (not on the causal pathway), namely infant age (chronological, i.e., not corrected for gestational age, modelled using splines with 5 knots)^32^; infant sex; calendar year of birth (categorical as individual years); mother age at delivery; mother’s WHO stage at delivery; whether the mother (re)initiated ART before pregnancy, during pregnancy, or during labour or later; mother’s education; mother’s occupation; mother’s marital status; and distance of residence from the clinic. Analyses were complete case, that is based on observed outcome measurements and those with missing data for any baseline variable were excluded from the adjusted models. In sensitivity analyses, we restricted to mothers who (re)initiated ART before the pregnancy; we considered 3 and 7 knot splines; we modelled calendar birth year as linear instead of categorical; we restricted the upper limit of the 18 month visit window from 30 to 21 months (further to request by peer reviewer); we restricted to infants born in 2019–2021 during which time both EFV and DTG-based maternal ART regimens were used (further to request by peer reviewer); and we performed inverse probability weighting analyses to account for drop out due to death, transfer out or lost to follow-up (further to request by peer reviewer; see Supplementary Methods for further details). In addition, we performed analyses stratified by infant sex. We used linear regression to evaluate factors associated with birthweight, among infants whose mothers initiated ART before or during pregnancy, adjusted for the same potential confounders as listed above plus mother’s weight at delivery and gestational age.

Analyses were performed using Stata version 16 and reporting followed STROBE guidelines.^33^^,^^34^

### Role of the funding source

The funders of the study had no role in study design; in the collection, analysis, and interpretation of data; in the writing of the report; nor in the decision to submit the paper for publication.

## Results

**Fig. 1:**
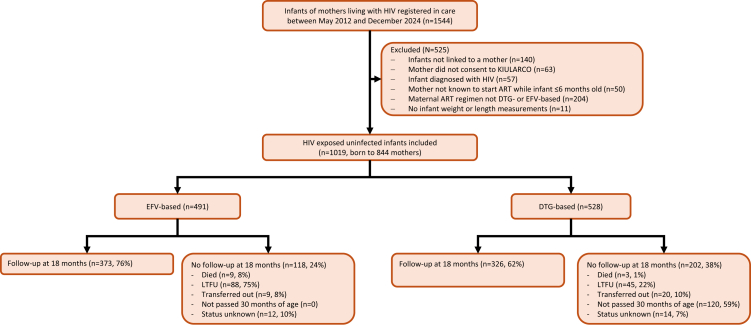
**Participant flowchart.** KIULARCO: Kilombero and Ulanga Antiretroviral Cohort; ART: antiretroviral therapy; DTG dolutegravir; EFV: efavirenz; LTFU: lost to follow-up.

Among 1544 infants registered in care at the CDCI, 525 were excluded (Fig. 1). Among the remaining 1019 infants born to 844 mothers, 491 (48%) mother–infant pairs were included in the EFV group and 528 (52%) in the DTG group. Overall, 165 (16%) of infants’ mothers switched ART during pregnancy; 45 (9%) in the EFV group and 120 (23%) in the DTG group (Table 1).

**Table 1:** Mother characteristics at delivery and pre-conception.

Mother characteristics	Maternal ART regimen		Total
	EFV group	DTG group	
Overall			
Number, n (row %)	491 (48%)	528 (52%)	1019 (100%)
ART regimen at delivery			
AZT+3TC+EFV	41 (8%)	0 (0%)	41 (4%)
TDF+FTC+EFV	29 (6%)	0 (0%)	29 (3%)
TDF+3TC+EFV	421 (86%)	0 (0%)	421 (41%)
ABC+3TC+DTG	0 (0%)	3 (1%)	3 (0%)
TDF+3TC+DTG	0 (0%)	524 (99%)	524 (51%)
AZT+3TC+DTG	0 (0%)	1 (0%)	1 (0%)
Switched ART regimen during pregnancy			
No	446 (91%)	408 (77%)	854 (84%)
Yes	45 (9%)	120 (23%)	165 (16%)
Age at delivery, years, median (IQR)	32 (26–36)	31 (26–36)	31 (26–36)
HIV WHO stage at delivery,^a^ n (%)			
I/II	325 (73%)	417 (82%)	742 (78%)
III/IV	122 (27%)	90 (18%)	212 (22%)
Missing	44 (9%)	21 (4%)	65 (6%)
Mothers who (re)initiated ART before the pregnancy:			
Number, n (row %)	260 (46%)	309 (54%)	569 (100%)
ART regimen at delivery			
AZT+3TC+EFV	34 (13%)	0 (0%)	34 (6%)
TDF+FTC+EFV	16 (6%)	0 (0%)	16 (3%)
TDF+3TC+EFV	210 (81%)	0 (0%)	210 (37%)
ABC+3TC+DTG	0 (0%)	3 (1%)	3 (1%)
TDF+3TC+DTG	0 (0%)	305 (99%)	305 (54%)
AZT+3TC+DTG	0 (0%)	1 (0%)	1 (0%)
Switched ART regimen during pregnancy			
No	222 (85%)	221 (72%)	443 (78%)
Yes	38 (15%)	88 (28%)	126 (22%)
Age at delivery, years, median (IQR)	33 (30–37)	33 (28–37)	33 (29–37)
HIV WHO stage at delivery,^a^ n (%)			
I/II	167 (64%)	232 (75%)	399 (70%)
III/IV	93 (36%)	76 (25%)	169 (30%)
Missing	0 (0%)	1 (0%)	1 (0%)
Time on ART at delivery, years, median (IQR)	3 (2–5)	5 (3–8)	4 (2–7)
Pre-conception BMI,^b^ n (%)			
Underweight (<18.5 kg/m^2^)	16 (6%)	10 (3%)	26 (5%)
Normal (18.5–<25 kg/m^2^)	149 (58%)	142 (49%)	291 (53%)
Overweight (25–<30 kg/m^2^)	67 (26%)	87 (30%)	154 (28%)
Obese (≥30 kg/m^2^)	24 (9%)	53 (18%)	77 (14%)
Missing	4 (2%)	17 (6%)	21 (4%)
Pre-conception CD4 count,^b^ cells/mm^3^, n (%)			
<200	10 (4%)	6 (5%)	16 (4%)
200–499	91 (40%)	34 (26%)	125 (35%)
≥500	127 (56%)	89 (69%)	216 (61%)
Missing	32 (12%)	180 (58%)	212 (37%)
Viral load at delivery,^c^ copies/ml, n (%)			
<100	85 (83%)	196 (96%)	281 (92%)
100–999	11 (11%)	4 (2%)	15 (5%)
≥1000	6 (6%)	5 (2%)	11 (4%)
Missing	158 (61%)	104 (34%)	262 (46%)
Mothers who (re)initiated ART during the pregnancy:			
Number, n (row %)	167 (49%)	172 (51%)	339 (100%)
ART regimen at delivery			
AZT+3TC+EFV	4 (2%)	0 (0%)	4 (1%)
TDF+FTC+EFV	8 (5%)	0 (0%)	8 (2%)
TDF+3TC+EFV	155 (93%)	0 (0%)	155 (46%)
TDF+3TC+DTG	0 (0%)	172 (100%)	172 (51%)
Switched ART regimen during pregnancy			
No	160 (96%)	140 (81%)	300 (88%)
Yes	7 (4%)	32 (19%)	39 (12%)
Age at delivery, years, median (IQR)	30 (25–34)	28 (23–33)	29 (24–33)
HIV WHO stage at delivery,^a^ n (%)			
I/II	130 (86%)	155 (93%)	285 (90%)
III/IV	22 (14%)	11 (7%)	33 (10%)
Missing	15 (9%)	6 (3%)	21 (6%)
Time on ART at delivery, months, median (IQR)	4 (2–6)	5 (3–8)	4 (2–7)
Viral load at delivery,^c^ copies/ml, n (%)			
<100	26 (84%)	116 (90%)	142 (89%)
100–999	2 (6%)	9 (7%)	11 (7%)
≥1000	3 (10%)	4 (3%)	7 (4%)
Missing	136 (81%)	43 (25%)	179 (53%)
Mothers who (re)initiated ART during labour or after delivery:			
Number, n (row %)	64 (58%)	47 (42%)	111 (100%)
ART regimen at delivery			
AZT+3TC+EFV	3 (5%)	0 (0%)	3 (3%)
TDF+FTC+EFV	5 (8%)	0 (0%)	5 (5%)
TDF+3TC+EFV	56 (88%)	0 (0%)	56 (50%)
TDF+3TC+DTG	0 (0%)	47 (100%)	47 (42%)
Switched ART regimen during pregnancy			
No	64 (100%)	47 (100%)	111 (100%)
Age at delivery, years, median (IQR)	29 (24–34)	29 (25–32)	29 (24–33)
HIV WHO stage at delivery,^a^ n (%)			
I/II	28 (80%)	30 (91%)	58 (85%)
III/IV	7 (20%)	3 (9%)	10 (15%)
Missing	29 (45%)	14 (30%)	43 (39%)

ART: antiretroviral therapy; EFV: efavirenz; DTG: dolutegravir; AZT: zidovudine; 3TC: lamivudine; TDF: tenofovir disoproxil fumarate; FTC: emtricitabine; ABC: abacavir; WHO: World Health Organization; BMI: body mass index; IQR: interquartile range. Of note, mothers who had multiple infants in the study are counted multiple times, once for each infant. Results are number and column percent of those with non-missing data except where otherwise indicated; missing data rows are number and column %.

a Closest result to delivery between enrolment into KIULARCO and up to 3 months after delivery.

b 21 to 9 months before delivery among non-pregnant women.

c +/−3 months relative to delivery.

At delivery, median maternal age was 31 years (interquartile range (IQR) 26–36) and 742 (78%) of mothers had a WHO stage I/II infection. Among the 569 (56%) mothers enrolled before pregnancy, the median time on ART at delivery was 4 years (IQR 2–7); pre-conception, 231 (42%) women were overweight or obese and 16 (4%) had CD4 cell counts <200/mm^3^; 11 (4%) women had a VL ≥ 1000 copies/ml at delivery. Among the 339 (33%) mothers enrolled during pregnancy, median time on ART at delivery was 4 months (IQR 2–7) and 7 (4%) had VL ≥1000 copies/ml at delivery. Maternal characteristics were broadly similar between the two ART groups, except that – compared to those in the EFV group – those in the DTG group were more likely to have lower WHO stage, be obese, have higher CD4 count, and have VL <100 copies/ml (for those with results). At KIULARCO enrolment, 787 (78% of those with non-missing data) women had primary education, 742 (74%) were farmers, 699 (69%) were married/cohabiting, and 623 (62%) lived within 1 km of the hospital (Supplementary Table S1).

Among infants, 526 (52%) were male, 968 (96% of those with non-missing data) were born at term, 984 (97%) at a health facility and 790 (78%) by non-instrumental vaginal delivery (Table 2). Median infant birthweight was 3.0 kg (IQR 2.7–3.3); 124 (13%) infants had birthweight <2.5 kg and 23 (2%) >4 kg. At registration, 727 (71%) infants were aged ≤6 weeks, 902 (98%) were on nevirapine prophylaxis and 203 (20%) on cotrimoxazole prophylaxis. Compared to the EFV group, there was a higher proportion of infants born by caesarean section, earlier registration to care after delivery, less frequent administration of cotrimoxazole prophylaxis, and fewer complete vaccinations among infants in the DTG group.

**Table 2:** Infant characteristics at birth and registration into care.

Infant characteristics	Maternal ART regimen		Total
	EFV group	DTG group	
Number (row %)	491 (48%)	528 (52%)	1019 (100%)
Birth year, n (%)			
2012	2 (0%)	0 (0%)	2 (0%)
2013	47 (10%)	0 (0%)	47 (5%)
2014	76 (15%)	0 (0%)	76 (7%)
2015	61 (12%)	0 (0%)	61 (6%)
2016	59 (12%)	0 (0%)	59 (6%)
2017	78 (16%)	0 (0%)	78 (8%)
2018	84 (17%)	0 (0%)	84 (8%)
2019	43 (9%)	58 (11%)	101 (10%)
2020	28 (6%)	98 (19%)	126 (12%)
2021	12 (2%)	107 (20%)	119 (12%)
2022	0 (0%)	87 (16%)	87 (9%)
2023	1 (0%)	87 (16%)	88 (9%)
2024	0 (0%)	91 (17%)	91 (9%)
Sex, n (%)			
Male	252 (51%)	274 (52%)	526 (52%)
Female	239 (49%)	254 (48%)	493 (48%)
Birthweight, kilograms, median (IQR)	3.0 (2.7–3.3)	3.0 (2.6–3.3)	3.0 (2.7–3.3)
<2.5 kg	56 (12%)	68 (13%)	124 (13%)
2.5–4 kg	411 (86%)	431 (84%)	842 (85%)
>4 kg	9 (2%)	14 (3%)	23 (2%)
Missing	15 (3%)	15 (3%)	30 (3%)
Gestational age at birth, n (%)			
Pre-term (<37 weeks)	12 (2%)	16 (3%)	28 (3%)
Term (37–42 weeks)	470 (97%)	498 (95%)	968 (96%)
Post-term (>42 weeks)	4 (1%)	8 (2%)	12 (1%)
Missing	5 (1%)	6 (1%)	11 (1%)
Place of birth, n (%)			
Health facility	469 (96%)	515 (98%)	984 (97%)
Home	19 (4%)	12 (2%)	31 (3%)
On the way to the health facility	3 (1%)	1 (0%)	4 (0%)
Method of delivery, n (%)			
Vaginal non-instrumented	410 (84%)	380 (72%)	790 (78%)
Caesarean section	74 (15%)	132 (25%)	206 (20%)
Vaginal instrumented	7 (1%)	16 (3%)	23 (2%)
Apgar score minute 1			
≥7	322 (98%)	384 (97%)	706 (97%)
<7	7 (2%)	13 (3%)	20 (3%)
Missing	162 (33%)	131 (25%)	293 (29%)
Apgar score minute 5			
≥7	329 (100%)	394 (99%)	723 (100%)
<7	0 (0%)	2 (1%)	2 (0%)
Missing	162 (33%)	132 (25%)	294 (29%)
Age at registration, weeks, n (%)			
0–6 weeks	312 (64%)	415 (79%)	727 (71%)
7–12 weeks	89 (18%)	61 (12%)	150 (15%)
13+ weeks	90 (18%)	52 (10%)	142 (14%)
Antiretroviral prophylaxis at registration, n (%)			
NVP	430 (100%)	472 (96%)	902 (98%)
ABC/3TC/LPV/r	2 (0%)	7 (1%)	9 (1%)
AZT/3TC/NVP	0 (0%)	13 (3%)	13 (1%)
Missing	59 (12%)	36 (7%)	95 (9%)
Cotrimoxazole prophylaxis status at registration, n (%)			
Started at registration	344 (71%)	395 (76%)	739 (73%)
On prior to registration	129 (26%)	74 (14%)	203 (20%)
Not given	14 (3%)	50 (10%)	64 (6%)
Missing	4 (1%)	9 (2%)	13 (1%)
BCG vaccination status at registration, n (%)			
Yes	463 (94%)	430 (81%)	893 (88%)
No	28 (6%)	98 (19%)	126 (12%)
Up to date on other scheduled vaccinations at registration, n (%)			
Yes	463 (94%)	446 (84%)	909 (89%)
No	28 (6%)	82 (16%)	110 (11%)

ART: antiretroviral therapy; EFV: efavirenz; DTG: dolutegravir; IQR: interquartile range; NVP: nevirapine; 3TC: lamivudine; LPV/r: lopinavir boosted with ritonavir; AZT: zidovudine; BCG: Bacillus Calmette-Guérin. Results are numbers and column percent of those with non-missing data except where otherwise indicated; missing data rows are number and column %.

Follow-up at 18 months was reached by 699 (69%) infants, 373 (76%) and 326 (62%) in the EFV and DTG groups, respectively. Of the remaining 320 infants, 12 died (9/118 (8%) in the EFV group and 3/202 (1%) in the DTG group), 133 were lost-to-follow-up (88 (75%) and 45 (22%), respectively), 29 were transferred out (9 (8%) and 20 (10%), respectively), 119 had not yet passed 30 months of age (closure of the 18-month window; all in the DTG group, 59% of 202), and status was unknown for 26 (12 (10%) in the EFV group and 14 (7%) in the DTG group). Exclusive breastfeeding was reported in 945/1002 (94%) infants shortly after birth, and at six months the corresponding result was 340/579 (59%; Supplementary Table S2). At 12 months, 436/488 (89%) infants had complementary feeding. Among 524 mothers with a VL measurement from birth up to 6 months of infant age, 461 (88%) had a VL <100 copies/ml, 34 (6%) had a VL of 100–999 copies/ml and 29 (6%) were unsuppressed with a VL ≥1000 copies/ml. Considering the results by the mother subgroups, VL during these first 6 months was ≥1000 copies/ml among 14/321 (4%) women enrolled before pregnancy, 8/163 (5%) women enrolled during pregnancy, and 7/40 (18%) women enrolled during labour or later.

**Fig. 2:**
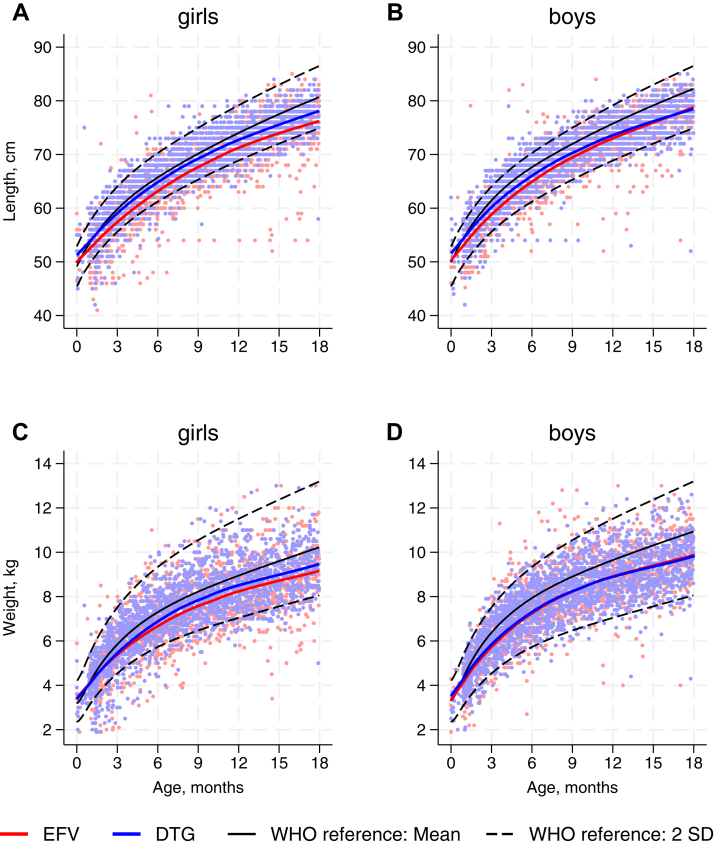
**Growth trajectories of HIV exposed uninfected infants by sex and maternal ART in comparison to WHO growth standards.** ART: antiretroviral therapy; EFV: efavirenz; DTG: dolutegravir; SD: standard deviation; WHO: World Health Organization. EFV and DTG indicates the maternal ART regimen (see Methods). WHO reference mean defines z-score of 0; WHO reference 2 SD by definition equates to z-score of ± 2 SD. Panels A and B show length against age for girls and boys, respectively, and C and D show weight against age for girls and boys, respectively.

**Fig. 3:**
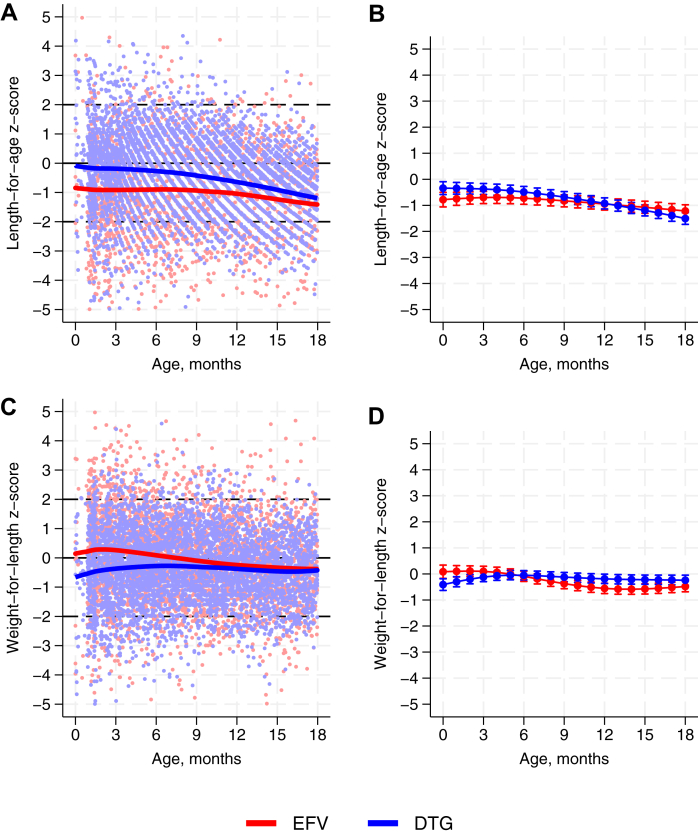
**Z-scores by maternal ART.** ART: antiretroviral therapy; EFV: efavirenz; DTG: dolutegravir. EFV and DTG indicates the maternal ART regimen (see Methods). Panels A and C are scatter plots of z-scores against infant age with running-line least squares smoothers. Scatter plot of length-for-age z-scores against age (A) omits 126 z-scores outside of the range −5 to +5 for clearer visualisation; similarly scatter plot of weight-for-length against age (C) omits 44 measurements. Panels B and D show marginal mean z-scores predicted over infant age (modelled using splines with 5 knots), based on random effects models adjusted for infant sex; calendar year of birth (categorical as individual years); mother’s age at delivery; mother’s WHO stage at delivery; whether mother (re)initiated ART before pregnancy versus during pregnancy versus during labour or breastfeeding; mother’s education; mother’s occupation; mother’s marital status; and distance of residence from the clinic. Error bars indicate 95% confidence intervals. Length-for-age z-score model (B) includes 8062 observations in 720 infants; weight-for-length z-score model (D) includes 8032 observations in 720 infants.

In total, 10,680 weight and 8585 length measurements were captured, in 1019 and 789 infants, respectively (Supplementary Fig. S1). Across both sexes and both EFV and DTG groups, infants had lower length and weight trajectories compared to WHO child growth standards (Fig. 2). Girls in the DTG group tended to have slightly higher lengths and weights compared to those in the EFV group, with no differences for boys. Translating the measurement to z-scores, infants in the DTG group tended to have higher length-for-age z-scores – but lower weight-for-length z-scores – at younger ages compared to infants in the EFV group, with little difference by 18 months (Fig. 3A and C, and Supplementary Fig. S2a). Models adjusted for confounders showed minimal differences between the two groups in terms of length-for-age and weight-for-length (Fig. 3B and D).

**Fig. 4:**
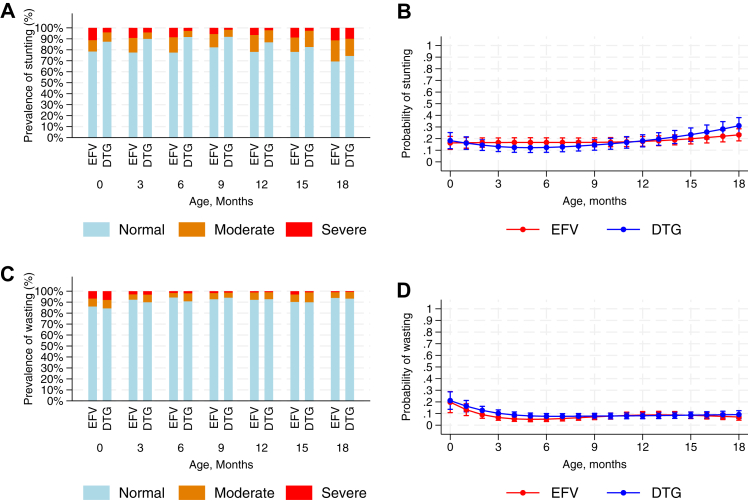
**Stunting and wasting by maternal ART.** ART: antiretroviral therapy; EFV: efavirenz; DTG: dolutegravir. EFV and DTG indicates the maternal ART regimen (see Methods). Panels A and C are bar graphs showing the proportions of infants with normal z-scores, moderate or severe stunting/wasting over time. Panels B and D show the predicted probabilities of being stunted/wasted (moderate and severe combined) over infant age (modelled using splines with 5 knots), based on logistic generalised estimating equations with exchangeable correlation structure, adjusted for infant sex; calendar year of birth (categorical as individual years); mother’s age at delivery; mother’s WHO stage at delivery; whether mother (re)initiated ART before pregnancy versus during pregnancy versus during labour or breastfeeding; mother’s education; mother’s occupation; mother’s marital status; and distance of residence from the clinic. Error bars indicate 95% confidence intervals. Stunting model (B) includes 8062 observations in 720 infants; wasting model (D) includes 8032 observations in 720 infants.

During follow-up, 1518/8585 (18%) visits indicated stunting and 713/8555 (8%) wasting, reflecting fluctuating z-scores over time with infants moving between normal and stunted/wasted categories over time. Overall, 377 infants were ever stunted (48% of 789 with at least one measurement) and 285 (36% of 789) ever wasted. In descriptive analyses, the prevalence of stunting was higher among those in the EFV group compared to the DTG group, ranging over visits during the first 18 months of life between 18 and 31% compared to 8–26%, respectively (Fig. 4A, and Supplementary Fig. S2b). However, after adjusting for confounders, there was no evidence of a difference between the groups (Fig. 4B). The prevalence of wasting was relatively low and comparable between the two groups across all time points (Fig. 4C and D).

There were no strong associations between the potential confounders and length-for-age or weight-for-length z-scores (Supplementary Table S3). Infants born to mothers who initiated ART during labour or breastfeeding were more likely to be stunted, compared to infants born to mothers who initiated ART before pregnancy, with no difference compared to those born to mothers who initiated during pregnancy. Infants born to mothers with later WHO stage were more likely to be wasted, while infants born to mothers with at least primary school education were less likely to be wasted, compared to no education. Results were robust to sensitivity analyses (Supplementary Fig. S2b–j) and stratification by infant sex (Supplementary Fig. S3a and b).

After adjustment for confounders, there was no evidence of a difference in birthweight between the two ART groups (adjusted mean difference 0.11 kg, 95% CI −0.03 to 0.26; Supplementary Table S4).

## Discussion

In this large cohort of HIV-exposed and uninfected infants, we found lower length and weight trajectories over 18 months compared to the WHO reference standards. In unadjusted analyses, there was some evidence of higher length-for-age z-scores and correspondingly lower risk of stunting, but lower weight-for-length z-scores, up to 6–12 months among infants born to mothers on DTG compared to EFV. However, there was no evidence of any differences by 18 months of age, nor in adjusted analyses. There was no evidence of a difference in the risk of wasting by maternal ART regimen. Infants were stunted at approximately 1 in 5 visits and wasted at approximately 1 in 10 visits.

The overall low length and weight trajectories of HEU – irrespective of maternal ART – are comparable to reports from similar settings. In a study of 624 HEU infants in Ethiopia, age and sex standardized weight and length were below the WHO reference at birth, and while weight-for-age normalised at 12 months, length-for-age worsened on average.^35^ In a randomised trial conducted in six African countries and India, mean length-for-age and weight-for-age z-scores in HEU were below the WHO reference up to two years of age.^36^ Further studies from Malawi, Uganda, Kenya, South Africa and Zambia confirmed the poor growth outcomes.^5^^,^^19^^,^^37^, ^38^, ^39^, ^40^, ^41^ In these studies, reasons for poorer growth outcome in HEU infants included poor maternal nutritional status, advanced HIV WHO stage, and poor social economic status.^5^^,^^19^^,^^35^, ^36^, ^37^, ^38^, ^39^, ^40^, ^41^

Over 18 months, we did not find a difference in the growth of HEU infants by maternal ART regimen. This is consistent with findings from a cohort of 1000 HEU children in Kenya, which did not find a difference in growth between infants exposed to DTG-, EFV- or protease inhibitor-based regimens.^19^ Further, the IMPAACT 2010 (VESTED) trial conducted in nine countries in Africa (including Tanzania), Asia and the Americas did not find a difference in weight-for-length between infants born to mothers randomised to DTG- versus EFV-based regimens. However, that trial found a difference in length-for-age, with infants born to mothers randomised to a DTG-based regimen having on average 0.4 higher z-score at 26 months and 0.3 higher at 50 weeks, compared to infants born to mothers randomised to an EFV-based regimen.^18^ Differences in the findings between studies may be attributable to the regional ethnical differences and related factors such as malnutrition and/or feeding practices including the length of breastfeeding, or a higher proportion of births at term or unmeasured confounding in our cohort.

Considering birthweight, we found no evidence of a difference by maternal ART regimen. This is in line with the DolPHIN-1 and DolPHIN-2 trials which also found no notable differences in birthweights of infants born to mothers on DTG versus EFV.^17^^,^^42^ Of note, the time on ART during pregnancy was notably shorter in the DolPHIN trials, where ART was initiated in the third trimester, compared to a large proportion of mothers in our study being on ART throughout pregnancy.

This study has limitations. Firstly, while reference can be made to WHO standards, we do not have a cohort of unexposed infants to enable direct comparison. Secondly, we cannot rule out unmeasured confounding, as DTG was rolled out in 2019 and therefore the mother–infant pairs in the EFV group were mainly captured pre-2019 and those in the DTG group from 2019 onwards. A sensitivity analysis restricting to enrolment years where infants were included in both the EFV and DTG groups showed similar findings to the primary analyses. However, the limited overlap meant that we could not perform additional analyses such as inverse probability weighting to account for ART assignment due to violation of the positivity assumption. Other changes over time, such as availability of food, might have affected maternal weight and infant growth, and such unmeasured confounding could have resulted in biased estimates. Thirdly, a third of mother–infant pairs did not complete 18-month follow-up, with a large proportion of those in the EFV group being lost to follow up and a large proportion of those in the DTG group not having yet reached the final follow-up, limiting the strength of evidence for longer-term outcomes. Fourthly, we were unable to account for mother’s pre-conception body mass index (BMI) which was missing for a notable proportion of women. Lastly, we included mother–infant pairs with at least one visit, meaning that there may be a selection bias and results cannot be generalised beyond those engaged in prevention of mother to child transmission (PMTCT) care and may underrepresent higher-risk infants (such as those born pre-term) who are at higher risk of morbidity and mortality.

In conclusion, in this large cohort-based study in rural Tanzania, we found no evidence of an association between maternal ART regimen and growth of HEU infants, but growth remained below WHO reference standards. Our findings support the continued use of DTG as the preferred first-line ART and emphasise the need for further research and targeted interventions to improve the health of the growing population of HIV-exposed uninfected infants.

## Contributors

Conceptualization: SG, MW, FV (lead), EL, GJM (supporting); data curation: JO, ED, MB, LW; formal analysis: FV (lead), JO, TG (supporting); funding acquisition: SG, MW (lead), FV (supporting); investigation: SG, EL, GS, EN, DM, LM, GJM; methodology: FV (lead), JO, FF, TG (supporting); project administration: MW (lead), SG, FV (supporting); supervision: DP, TG, MW, FV; validation: JO, FV; visualization: JO, FV; writing – original draft: SG, EL, FV; writing – review & editing: all co-authors. JO and FV directly accessed and verified the underlying data reported in the manuscript. All authors confirm that they had full access to all the data in the study and accept responsibility to submit for publication.

## Data sharing statement

A subset of the key pseudo-anonymised individual participant data from the study, along with a data dictionary, is available upon request through the data repository Zenodo (DOI 10.5281/zenodo.20271866).

## Declaration of interests

All authors declare no conflict of interests, except Maja Weisser received travel grants for ESSCMID Global and IAS conferences from Gilead and Pfizer.

## Appendix A Supplementary data

Supplementary information

Translated abstract
