# Supplementary material for: The impact of dolutegravir on the growth of HIV-exposed uninfected infants: an observational cohort study in rural Tanzania
Source: eClinicalMedicine. 2026 May 28;96:103984. doi: 10.1016/j.eclinm.2026.103984 (PMC13233611; doi:10.1016/j.eclinm.2026.103984)
Supplement: Supplementary information [file mmc1.docx]

**Supplementary material**

Contents

KIULARCO Study Group 2

[Supplementary Methods 3](#_Toc226124455)

[Study procedures 3](#_Toc226124456)

[Statistical methods 3](#_Toc226124457)

[Supplementary Table 1. Maternal characteristics at enrolment into KIULARCO. 5](#_Toc226124458)

[Supplementary Table 2. Infant feeding. 6](#_Toc226124459)

[Supplementary Table 3. Results from growth models. 7](#_Toc226124460)

[Supplementary Table 4. Birth weight model results. 9](#_Toc226124461)

[Supplementary Figure 1. Weight versus height by sex. 10](#_Toc226124462)

[Supplementary Figure 2a. Sensitivity analysis: unadjusted models. 11](#_Toc226124463)

[Supplementary Figure 2b. Sensitivity analysis: restricting to mothers who (re)initiated ART before pregnancy. 12](#_Toc226124464)

[Supplementary Figure 2c. Sensitivity analysis: 3 knots. 13](#_Toc226124465)

[Supplementary Figure 2d. Sensitivity analysis: 7 knots. 14](#_Toc226124466)

[Supplementary Figure 2e. Sensitivity analysis: with adjustment for birth year under linearity assumption. 15](#_Toc226124467)

[Supplementary Figure 2f. Sensitivity analysis: restriction of upper limit of 18 month visit window from 30 to 21 months. 16](#_Toc226124468)

[Supplementary Figure 2g. Sensitivity analysis: adjustment for whether mother switched ART regimen during pregnancy. 17](#_Toc226124469)

[Supplementary Figure 2h. Sensitivity analysis: restriction to mothers who did not switch ART regimen during pregnancy. 18](#_Toc226124470)

[Supplementary Figure 2i. Sensitivity analysis: restricting to birth years where both maternal ART regimens containing EFV or DTG were used (2019-2021). 19](#_Toc226124471)

[Supplementary Figure 2j. Sensitivity analysis: accounting for drop out through inverse probability weighting. 20](#_Toc226124472)

[Supplementary Figure 3a. Stratification by sex: z-scores. 21](#_Toc226124473)

[Supplementary Figure 3b. Stratification by sex: stunting and wasting. 22](#_Toc226124474)

[References 23](#_Toc226124475)

**KIULARCO Study Group**

Aschola Asantiel, Farida Bani, Manuel Battegay, Mathias E Bukuku, Theonestina Byakuzana, Adolphina Chale, Joyce Claud, Elizabeth K Dotto, Kabula A Elias , Gideon Francis, Tracy Glass, Yvonne Haridas, Speciosa Hwaya, Gift Joseph, Rodney M Julius, Fatuma Kabelele, Aneth V Kalinjuma, Andrew Katende, Fredrick Kazetera, Yassin Kisunga, Bernard Kivuma, Thomas Klimkait, Juma Kupewa, Ezekiel Luoga, Jerome Lwali, Clarence A Mahundo, Mkiwa A Makwea, Edgar E Martin, Honorati Masanja, Swalehe Masoud, Efrazia Mayanzani, Mohammed Mbaruku, Geofrey Mbunda, George P Mfanando, Josephine Mhina, Mengi Mkulila, Margareth Mkusa, Christina Mluge, Franzisca A Mmbando, Alpha Mninje, Lina Mnunga, Dorcas K Mnzana, Getrud J Mollel, Lilian Moshi, Germana Mossad, Regina Mponji, Dolores Mpundunga, Athumani Mtandanguo, Ummu-kulthum Mwaliga, Alice I Mwanga, Sanula Nahota, Sharifa Nakapala, Regina Ndaki, Robert C Ndege, Happyphania Ngakongwa, Agatha Ngulukila, Alex John Ntamatungiro, Emmanuel Nyenza, James Okuma, Ally Olotu, Daniel H Paris , Albert Raphael, Martin Rohacek, Leila Samson, Elizabeth Senkoro, George Sigalla, Jamali B Siru , Jenifa Tarimo, Juerg Utzinger, Fiona Vanobberghen, Maja Weisser, John Wigay, Lulu Wilson

# **Supplementary Methods**

## Study procedures

#### At clinic visits, vaccination status is assessed (WHO Vaccination Schedule for Tanzania);^1^ post-partum ART prophylaxis (nevirapine for low-risk infants and combination therapy for high-risk infants) is initiated <4 weeks after birth; cotrimoxazole preventive therapy is initiated at 6 weeks of age until HIV infection has been ruled out or cessation of breastfeeding; infant feeding practices are recommended (exclusive breastfeeding until 6 months, followed by complementary feeding until 12 months, after which breastfeeding should be stopped);^2^ hospital admissions, current complaints, new comorbidities are captured; and HIV testing is performed (HIV DNA PCR or rapid antibody testing at 6 weeks and 9 months after birth and 3 months after cessation of breastfeeding depending on guidelines at the time, and rapid antibody testing at 18 months).^2^ Maternal viral load (VL) is tested three months after ART initiation in newly-diagnosed, pregnant and breastfeeding women, and at report of pregnancy for those who are ART-experienced; further VL testing is done no later than 3 months before delivery, and six-monthly until cessation of breastfeeding.^2^ Women with VL >50 copies/mL (>1000 copies/mL pre-2019) undergo enhanced adherence counselling for three months followed by repeat VL testing.^2^ Within routine care, if a patient misses an appointment, they are called within 3 days. In addition, professional counsellors perform regular individual and group counselling during pregnancy and after delivery to emphasise the importance of ART intake and visit attendance. Further, consenting pregnant and breastfeeding women are called 2-3 days before their clinical visits as a reminder. There is an additional option for mother-to-mother peer support.

## Statistical methods

For mothers enrolled in KIULARCO before or during pregnancy, we described time on ART and VL at time of delivery (+/- 3 months) and additionally preconception body mass index (BMI) and CD4 count (latest values between 21 weeks and 9 months before delivery) in those on ART before pregnancy. Infants’ characteristics were summarised at delivery – including neonatal APGAR (Appearance, Pulse, Grimace, Activity and Respiration) scores at one and five minutes (values of ≥7 were considered normal)^3^ – and at registration into postnatal care.

In sensitivity analyses, we performed inverse probability weighting to account for drop out (death, transfer out or lost to follow up). Firstly, we fit a logistic regression model with outcome drop out and independent variables as for the primary analyses, plus the ART group (DTG versus EFV), birth weight, gestational age, delivery place, delivery method and infant age at registration. From this model, we obtained weights as the inverse probability of drop out. Finally, we fit the same outcome models as for the primary analysis, incorporating these weights.

# **Supplementary** Table 1. Maternal characteristics at enrolment into KIULARCO.

| **Maternal characteristics** | **Maternal ART regimen** | | **Total** |
| --- | --- | --- | --- |
|  | **EFV group** | **DTG group** |  |
| Number (%) | 491 (48%) | 528 (52%) | 1,019 (100%) |
| Calendar year, n (%) |  |  |  |
| 2005-2012 | 161 (33%) | 80 (15%) | 241 (24%) |
| 2013-2016 | 226 (46%) | 85 (16%) | 311 (31%) |
| 2017-2025 | 104 (21%) | 363 (69%) | 467 (46%) |
| Age, years, median (IQR) | 29 (25-33) | 28 (24-31) | 28 (24-32) |
| Highest education, n (%) |  |  |  |
| None | 34 (7%) | 34 (6%) | 68 (7%) |
| Primary school | 398 (82%) | 389 (74%) | 787 (78%) |
| Secondary and above | 53 (11%) | 101 (19%) | 154 (15%) |
| Missing | 6 (1%) | 4 (1%) | 10 (1%) |
| Occupation, n (%) |  |  |  |
| Farmer | 386 (80%) | 356 (68%) | 742 (74%) |
| Non-Farmer | 99 (20%) | 168 (32%) | 267 (26%) |
| Missing | 6 (1%) | 4 (1%) | 10 (1%) |
| Marital status, n (%) |  |  |  |
| Married/Cohabiting | 337 (69%) | 362 (69%) | 699 (69%) |
| Never married | 58 (12%) | 84 (16%) | 142 (14%) |
| Separated/divorced/widowed | 95 (19%) | 82 (16%) | 177 (17%) |
| Missing | 1 (0%) | 0 (0%) | 1 (0%) |
| Number of children, median (IQR) | 2 (1-3) | 2 (1-3) | 2 (1-3) |
| Distance of residence from clinic, n (%) |  |  |  |
| **≤** 1km | 275 (57%) | 348 (67%) | 623 (62%) |
| 2 - <50km | 138 (28%) | 128 (25%) | 266 (27%) |
| ≥ 50km | 72 (15%) | 41 (8%) | 113 (11%) |
| Missing | 6 (1%) | 11 (2%) | 17 (2%) |

EFV: efavirenz; DTG: dolutegravir; ART: antiretroviral therapy; IQR: interquartile range. Of note, mothers who had multiple infants in the study are counted multiple times, once for each infant. Results are number and column percent of those with non-missing data except where otherwise indicated; missing data rows are number and column %.

# **Supplementary** Table 2. Infant feeding.

| **Age, months** | **Infant feeding** | **Maternal ART regimen** | | **Total** |
| --- | --- | --- | --- | --- |
|  |  | **EFV** | **DTG** |  |
| 0 | Exclusive breastfeeding | 445 (92%) | 500 (97%) | 945 (94%) |
|  | Formula milk | 7 (1%) | 7 (1%) | 14 (1%) |
|  | Animal milk | 7 (1%) | 0 (0%) | 7 (1%) |
|  | Mixed or complementary feeding | 25 (5%) | 11 (2%) | 36 (4%) |
| 3 | Exclusive breastfeeding | 313 (94%) | 242 (98%) | 555 (95%) |
|  | Formula milk | 5 (2%) | 2 (1%) | 7 (1%) |
|  | Animal milk | 3 (1%) | 0 (0%) | 3 (1%) |
|  | Mixed or complementary feeding | 13 (4%) | 4 (2%) | 17 (3%) |
| 6 | Exclusive breastfeeding | 199 (55%) | 141 (64%) | 340 (59%) |
|  | Formula milk | 9 (3%) | 5 (3%) | 14 (2%) |
|  | Animal milk | 6 (2%) | 0 (0%) | 6 (1%) |
|  | Mixed or complementary feeding | 145 (40%) | 74 (34%) | 219 (38%) |
| 9 | Exclusive breastfeeding | 35 (11%) | 12 (6%) | 47 (9%) |
|  | Formula milk | 6 (2%) | 0 (0%) | 6 (1%) |
|  | Animal milk | 1 (1%) | 1 (1%) | 2 (<1%) |
|  | Mixed or complementary feeding | 282 (87%) | 172 (93%) | 454 (89%) |
| 12 | Exclusive breastfeeding | 29 (10%) | 9 (5%) | 38 (8%) |
|  | Formula milk | 5 (2%) | 0 (0%) | 5 (1%) |
|  | Animal milk | 2 (1%) | 7 (4%) | 9 (2%) |
|  | Mixed or complementary feeding | 263 (88%) | 173 (92%) | 436 (89%) |
| 15 | Exclusive breastfeeding | 25 (9%) | 10 (6%) | 35 (8%) |
|  | Formula milk | 1 (<1%) | 0 (0%) | 1 (<1%) |
|  | Animal milk | 2 (1%) | 5 (3%) | 7 (2%) |
|  | Mixed or complementary feeding | 249 (90%) | 157 (91%) | 406 (90%) |
| 18 | Exclusive breastfeeding | 17 (6%) | 12 (7%) | 29 (6%) |
|  | Formula milk | 3 (1%) | 0 (0%) | 3 (1%) |
|  | Animal milk | 4 (2%) | 5 (3%) | 9 (2%) |
|  | Mixed or complementary feeding | 282 (92%) | 166 (91%) | 448 (92%) |

EFV: efavirenz; DTG: dolutegravir; ART: antiretroviral therapy. **Results are number (column % of the infants with data captured at each respective visit).**

# **Supplementary** Table 3. Results from growth models.

| **Outcome:** | **Length-for-age z-score** | | **Weight-for-length z-score** | | **Stunting** | | **Wasting** | |
| --- | --- | --- | --- | --- | --- | --- | --- | --- |
| Sex, versus male |  |  |  |  |  |  |  |  |
| Female | 0.08 | [-0.11; 0.27] | 0.05 | [-0.11; 0.21] | 0.84 | [0.65; 1.08] | 0.84 | [0.63; 1.12] |
| Birth year, versus 2013 |  |  |  |  |  |  |  |  |
| 2014 | -0.39 | [-0.91; 0.14] | 0.14 | [-0.30; 0.58] | 1.53 | [0.82; 2.84] | 0.50 | [0.20; 1.21] |
| 2015 | -0.45 | [-0.99; 0.10] | -0.18 | [-0.64; 0.27] | 1.72 | [0.91; 3.26] | 1.16 | [0.51; 2.63] |
| 2016 | 0.28 | [-0.27; 0.83] | 0.10 | [-0.36; 0.56] | 0.57 | [0.28; 1.14] | 0.59 | [0.24; 1.44] |
| 2017 | 0.29 | [-0.27; 0.85] | -0.22 | [-0.69; 0.24] | 0.54 | [0.26; 1.10] | 0.92 | [0.40; 2.09] |
| 2018 | 0.04 | [-0.48; 0.56] | -0.06 | [-0.50; 0.37] | 0.73 | [0.38; 1.39] | 0.77 | [0.35; 1.69] |
| 2019 | -0.03 | [-0.59; 0.53] | -0.45 | [-0.92; 0.02] | 0.85 | [0.42; 1.74] | 1.16 | [0.51; 2.64] |
| 2020 | 0.36 | [-0.21; 0.93] | -0.48 | [-0.95; -0.00] | 0.33 | [0.15; 0.73] | 0.81 | [0.34; 1.90] |
| 2021 | 0.70 | [0.11; 1.30] | -0.66 | [-1.15; -0.16] | 0.31 | [0.14; 0.71] | 0.87 | [0.36; 2.08] |
| 2022 | 0.51 | [-0.17; 1.20] | -0.80 | [-1.37; -0.24] | 0.24 | [0.09; 0.65] | 0.89 | [0.33; 2.40] |
| 2023 | 0.11 | [-0.55; 0.77] | -0.55 | [-1.10; -0.00] | 0.59 | [0.25; 1.43] | 0.87 | [0.33; 2.26] |
| 2024 | 0.09 | [-0.55; 0.72] | -0.65 | [-1.18; -0.12] | 0.60 | [0.25; 1.46] | 0.87 | [0.34; 2.23] |
| Maternal age at delivery, per year | -0.00 | [-0.02; 0.02] | -0.01 | [-0.02; 0.00] | 0.98 | [0.96; 1.01] | 1.00 | [0.97; 1.02] |
| WHO stage, versus I/II |  |  |  |  |  |  |  |  |
| III/IV | -0.03 | [-0.26; 0.21] | -0.09 | [-0.28; 0.11] | 1.16 | [0.85; 1.60] | 1.69 | [1.21; 2.37] |
| When mother (re)initiated ART, versus before pregnancy versus |  |  |  |  |  |  |  |  |
| During pregnancy | 0.00 | [-0.22; 0.22] | -0.01 | [-0.20; 0.17] | 0.99 | [0.73; 1.35] | 1.36 | [0.97; 1.90] |
| During labour or breastfeeding | -0.24 | [-0.62; 0.15] | -0.22 | [-0.55; 0.10] | 1.93 | [1.21; 3.10] | 1.41 | [0.80; 2.48] |
| Maternal highest education, versus None |  |  |  |  |  |  |  |  |
| Primary school | 0.24 | [-0.14; 0.62] | 0.02 | [-0.29; 0.34] | 0.65 | [0.40; 1.07] | 0.56 | [0.35; 0.90] |
| Secondary and above | 0.15 | [-0.32; 0.61] | 0.31 | [-0.08; 0.69] | 0.65 | [0.35; 1.21] | 0.41 | [0.22; 0.76] |
| Maternal occupation, versus non-farmer |  |  |  |  |  |  |  |  |
| Farmer | -0.02 | [-0.25; 0.22] | 0.02 | [-0.18; 0.21] | 0.92 | [0.67; 1.26] | 1.12 | [0.79; 1.58] |
| Maternal marital status, versus married |  |  |  |  |  |  |  |  |
| Never married | -0.23 | [-0.50; 0.04] | -0.00 | [-0.23; 0.23] | 1.32 | [0.92; 1.90] | 1.34 | [0.92; 1.95] |
| Separated/ divorced/ widowed | -0.24 | [-0.50; 0.01] | 0.16 | [-0.05; 0.38] | 1.14 | [0.81; 1.62] | 0.66 | [0.43; 1.02] |
| Distance of residence from clinic, versus <2 km |  |  |  |  |  |  |  |  |
| 2-<50km | 0.13 | [-0.10; 0.35] | 0.10 | [-0.09; 0.28] | 0.91 | [0.67; 1.24] | 0.75 | [0.53; 1.05] |
| >=50km | 0.15 | [-0.18; 0.48] | 0.18 | [-0.10; 0.46] | 0.88 | [0.56; 1.40] | 0.59 | [0.32; 1.09] |

EFV: efavirenz; DTG: dolutegravir; ART: antiretroviral therapy; WHO: World Health Organization. **Models for length-for-age and weight-for-length z-scores are random effects models, and for stunting and wasting are logistic generalised estimating equations models. Reported results for each variable are estimate and 95% confidence interval. Results for ART group (DTG vs EFV) and infant age (modelled using 5-knot splines) are omitted from this table and instead illustrated in Figures 3 and 4.**

# **Supplementary** Table 4. Birth weight model results.

|  | **Estimate** | **95% confidence interval** |
| --- | --- | --- |
| ART group, versus EFV |  |  |
| DTG | 0.11 | [-0.03; 0.26] |
| Sex, versus male |  |  |
| Female | -0.09 | [-0.16; -0.02] |
| Gestational age, versus Term (37-42 weeks) |  |  |
| Pre-term (<37 weeks) | -0.91 | [-1.15; -0.67] |
| Post-term (>42 weeks) | 0.47 | [0.12; 0.82] |
| Birth year, versus 2012 |  |  |
| 2013 | 0.33 | [-0.66; 1.32] |
| 2014 | 0.19 | [-0.80; 1.18] |
| 2015 | 0.27 | [-0.72; 1.25] |
| 2016 | 0.28 | [-0.71; 1.27] |
| 2017 | 0.36 | [-0.63; 1.34] |
| 2018 | 0.29 | [-0.69; 1.28] |
| 2019 | 0.22 | [-0.76; 1.21] |
| 2020 | 0.11 | [-0.87; 1.10] |
| 2021 | 0.24 | [-0.74; 1.23] |
| 2022 | 0.20 | [-0.79; 1.19] |
| 2023 | 0.14 | [-0.85; 1.14] |
| 2024 | 0.04 | [-0.96; 1.03] |
| Maternal age at delivery, per year | -0.00 | [-0.01; 0.00] |
| WHO stage at delivery, versus I/II |  |  |
| III/IV | -0.00 | [-0.09; 0.09] |
| When mother (re)initiated ART, versus before pregnancy versus |  |  |
| During pregnancy | -0.04 | [-0.12; 0.04] |
| Maternal highest education, versus None |  |  |
| Primary school | -0.04 | [-0.18; 0.11] |
| Secondary and above | -0.03 | [-0.20; 0.14] |
| Maternal occupation, versus non-farmer |  |  |
| Farmer | 0.02 | [-0.07; 0.11] |
| Maternal marital status, versus married |  |  |
| Never married | -0.12 | [-0.22; -0.01] |
| Separated/ divorced/ widowed | -0.07 | [-0.17; 0.03] |
| Distance of residence from clinic, versus <2 km |  |  |
| 2-<50km | 0.09 | [0.00; 0.17] |
| >=50km | 0.08 | [-0.04; 0.20] |
| Mother weight at delivery, kg [1] | 0.01 | [0.01; 0.01] |

EFV: efavirenz; DTG: dolutegravir; ART: antiretroviral therapy; WHO: World Health Organization. **Results from linear regression model. [1] First measurement between birth and up to maximum six months later.**

# **Supplementary** Figure 1. Weight versus height by sex.

EFV: efavirenz; DTG: dolutegravir. Graphs show running-line least squares smoother.

# **Supplementary** Figure 2a. Sensitivity analysis: unadjusted models.

EFV: efavirenz; DTG: dolutegravir. Graphs in upper row show marginal mean z-scores predicted over infant age (modelled using splines with 5 knots), based on random effects models. Graphs in lower row show probability of stunting or wasting predicted from logistic generalised estimating equations with exchangeable correlation structure with infant age modelled using splines with 5 knots. Error bars are 95% confidence intervals. Length-for-age z-score model includes 8585 observations in 789 infants; weight-for-length z-score model includes 8555 observations in 789 infants; stunting model includes 8585 observations in 789 infants; wasting model includes 8585 observations in 789 infants.

# Supplementary Figure 2b. Sensitivity analysis: restricting to mothers who (re)initiated ART before pregnancy.

EFV: efavirenz; DTG: dolutegravir; ART: antiretroviral therapy. Graphs in upper row show marginal mean z-scores predicted over infant age (modelled using splines with 5 knots), based on random effects models. Graphs in lower row show probability of stunting or wasting predicted from logistic generalised estimating equations with exchangeable correlation structure with infant age modelled using splines with 5 knots. Adjustment as for primary analysis. Error bars are 95% confidence intervals. Length-for-age z-score model includes 5029 observations in 427 infants; weight-for-length z-score model includes 5009 observations in 427 infants; stunting model includes 5029 observations in 427 infants; wasting model includes 5009 observations in 427 infants.

# **Supplementary Figure 2c. Sensitivity analysis: 3 knots.**

 EFV: efavirenz; DTG: dolutegravir. Graphs in upper row show marginal mean z-scores predicted over infant age (modelled using splines with 3 knots), based on random effects models. Graphs in lower row show probability of stunting or wasting predicted from logistic generalised estimating equations with exchangeable correlation structure with infant age modelled using splines with 3 knots. Adjustment and numbers of observations as per primary analysis. Error bars are 95% confidence intervals.

# **Supplementary Figure 2d. Sensitivity analysis: 7 knots.**

 EFV: efavirenz; DTG: dolutegravir. Graphs in upper row show marginal mean z-scores predicted over infant age (modelled using splines with 7 knots), based on random effects models. Graphs in lower row show probability of stunting or wasting predicted from logistic generalised estimating equations with exchangeable correlation structure with infant age modelled using splines with 7 knots. Adjustment and numbers of observations as per primary analysis. Error bars are 95% confidence intervals.

# **Supplementary Figure 2e. Sensitivity analysis: with adjustment for birth year under linearity assumption.**

EFV: efavirenz; DTG: dolutegravir. Graphs in upper row show marginal mean z-scores predicted over infant age (modelled using splines with 5 knots), based on random effects models. Graphs in lower row show probability of stunting or wasting predicted from logistic generalised estimating equations with exchangeable correlation structure with infant age modelled using splines with 5 knots. Adjustment and numbers of observations as per primary analysis, except with birth year included as linear variable. Error bars are 95% confidence intervals.

# **Supplementary Figure 2f. Sensitivity analysis: restriction of upper limit of 18 month visit window from 30 to 21 months.**

EFV: efavirenz; DTG: dolutegravir. Graphs in upper row show marginal mean z-scores predicted over infant age (modelled using splines with 5 knots), based on random effects models. Graphs in lower row show probability of stunting or wasting predicted from logistic generalised estimating equations with exchangeable correlation structure with infant age modelled using splines with 5 knots. Adjustment as for primary analysis. Error bars are 95% confidence intervals. Length-for-age z-score model includes 7748 observations in 719 infants; weight-for-length z-score model includes 7727 observations in 719 infants; stunting model includes 7748 observations in 719 infants; wasting model includes 7727 observations in 719 infants.

# **Supplementary Figure 2g. Sensitivity analysis: adjustment for whether mother switched ART regimen during pregnancy.**

EFV: efavirenz; DTG: dolutegravir; ART: antiretroviral therapy. Graphs in upper row show marginal mean z-scores predicted over infant age (modelled using splines with 5 knots), based on random effects models. Graphs in lower row show probability of stunting or wasting predicted from logistic generalised estimating equations with exchangeable correlation structure with infant age modelled using splines with 5 knots. Adjustment and numbers of observations as for primary analysis, except also including an indicator for whether mother switched ART regimen during pregnancy. Error bars are 95% confidence intervals.

# **Supplementary Figure 2h. Sensitivity analysis: restriction to mothers who did not switch ART regimen during pregnancy.**

EFV: efavirenz; DTG: dolutegravir; ART: antiretroviral therapy. Graphs in upper row show marginal mean z-scores predicted over infant age (modelled using splines with 5 knots), based on random effects models. Graphs in lower row show probability of stunting or wasting predicted from logistic generalised estimating equations with exchangeable correlation structure with infant age modelled using splines with 5 knots. Adjustment as for primary analysis. Error bars are 95% confidence intervals. Length-for-age z-score model includes 6683 observations in 599 infants; weight-for-length z-score model includes 6659 observations in 599 infants; stunting model includes 6683 observations in 599 infants; wasting model includes 6659 observations in 599 infants.

# **Supplementary Figure 2i. Sensitivity analysis: restricting to birth years where both maternal ART regimens containing EFV or DTG were used (2019-2021).**

EFV: efavirenz; DTG: dolutegravir. Graphs in upper row show marginal mean z-scores predicted over infant age (modelled using splines with 5 knots), based on random effects models. Graphs in lower row show probability of stunting or wasting predicted from logistic generalised estimating equations with exchangeable correlation structure with infant age modelled using splines with 5 knots. Adjustment as for primary analysis. Error bars are 95% confidence intervals. Length-for-age z-score model includes 3054 observations in 255 infants; weight-for-length z-score model includes 3050 observations in 255 infants; stunting model includes 3054 observations in 255 infants; wasting model includes 3050 observations in 255 infants.

# **Supplementary Figure 2j. Sensitivity analysis: accounting for drop out through inverse probability weighting.**

EFV: efavirenz; DTG: dolutegravir. Graphs in upper row show marginal mean z-scores predicted over infant age (modelled using splines with 5 knots), based on random effects models. Graphs in lower row show probability of stunting or wasting predicted from logistic generalised estimating equations with exchangeable correlation structure with infant age modelled using splines with 5 knots. Adjustment as for primary analysis, incorporating inverse probability weighting for drop out. Error bars are 95% confidence intervals.

# **Supplementary Figure 3a. Stratification by sex: z-scores.**

EFV: efavirenz; DTG: dolutegravir. Graphs in upper row show marginal mean z-scores predicted over infant age (modelled using splines with 5 knots), based on random effects models. Graphs in lower row show probability of stunting or wasting predicted from logistic generalised estimating equations with exchangeable correlation structure with infant age modelled using splines with 5 knots. Adjustment as for primary analysis. Error bars are 95% confidence intervals. For boys: length-for-age z-score model includes 4134 observations in 371 infants; weight-for-length z-score model includes 4126 observations in 371 infants. For girls: length-for-age z-score model includes 3928 observations in 349 infants; weight-for-length z-score model includes 3906 observations in 349 infants.

# **Supplementary Figure 3b. Stratification by sex: stunting and wasting.**

EFV: efavirenz; DTG: dolutegravir. Graphs in upper row show marginal mean z-scores predicted over infant age (modelled using splines with 5 knots), based on random effects models. Graphs in lower row show probability of stunting or wasting predicted from logistic generalised estimating equations with exchangeable correlation structure with infant age modelled using splines with 5 knots. Adjustment as for primary analysis. Error bars are 95% confidence intervals. For boys: length-for-age z-score model includes 4134 observations in 371 infants; weight-for-length z-score model includes 4126 observations in 371 infants. For girls: length-for-age z-score model includes 3928 observations in 349 infants; weight-for-length z-score model includes 3906 observations in 349 infants.

# **References**

1. Ministry Of Health And Social Welfare – Tanzania Mainland. Expanded programme on immunization 2010-2015 comprehensive multi year plan. Published online 2011. Accessed October 14, 2025. https://extranet.who.int/countryplanningcycles/sites/default/files/planning_cycle_repository/tanzania/tanzania_cmyp_doc.pdf

2. Ministry of Health, Community Development, Gender, Elderly, and Children. National Guidelines for the Management of HIV and AIDS; 7th edition. Published online 2019.

3. Committee on Obstetric Practice. The Apgar Score. Published online October 2015. Accessed June 19, 2025. https://www.acog.org/-/media/project/acog/acogorg/clinical/files/committee-opinion/articles/2015/10/the-apgar-score.pdf?rev=a77f52bbff2144c9b52cb1c986f0947f&hash=D1A8CD40B6C7B3252EF7C0E1A20AA3AAhttps://www.acog.org/-/media/project/acog/acogorg/clinical/files/committee-opinion/articles/2015/10/the-apgar-score.pdf?rev=a77f52bbff2144c9b52cb1c986f0947f&hash=D1A8CD40B6C7B3252EF7C0E1A20AA3AA
