# Supplementary material for: The impact of dolutegravir on the growth of HIV-exposed uninfected infants: an observational cohort study in rural Tanzania
Source: eClinicalMedicine. 2026 May 28;96:103984. doi: 10.1016/j.eclinm.2026.103984 (PMC13233611; doi:10.1016/j.eclinm.2026.103984)
Supplement: Translated abstract [file mmc2.docx]

**The impact of dolutegravir on the growth of HIV-exposed uninfected infants: an observational cohort study in rural Tanzania**

**Athari za dolutegravir katika ukuaji wa watoto wachanga waliokuwa kwenye mazingira ya VVU bila kuambukizwa: utafiti wa kundi la uangalizi vijijini mwaTanzania**

**Sara Glanzmann, MD^1^*, Ezekiel Luoga, MD^2,3^*, James Okuma, MSc^4,5^, Elizabeth Dotto, BSc^2^, George Sigalla^2,3^, Fabian Christoph Franzeck, MD^5,6,7^,** Emanuel Nyenza**, MD^2,3^, Dorcas Mnzava, MSc^2,8^, Mathias Bukuku, BSc^2^, Lilian Moshi, MD^2,3^, Lulu Wilson, MSc^2^, Daniel H. Paris, MD^4,5^, Tracy R. Glass, PhD^4,5^, Getrud Joseph Mollel, MD^2,3,9^, Maja Weisser, MD^1,2,4,5,^°, Fiona Vanobberghen, PhD^4,5^° on behalf of the KIULARCO Study Group**

**^1^ Division of Infectious Diseases, University Hospital Basel, Basel, Switzerland**

**^2^ Ifakara Health Institute, Ifakara, Tanzania**

**^3^ St. Francis Regional Referral Hospital, Ifakara, Tanzania**

**^4^ Swiss Tropical and Public Health Institute, Allschwil, Switzerland**

**^5^ University of Basel, Basel, Switzerland**

**^6^** Research and analytics, Department of Informatics, University Hospital Basel, Basel

**^7^ Department of Infectious Diseases, University Medical Center Basel-Land, Liestal, Switzerland**

**^8^ Charité – University Medicine Berlin, Berlin, Germany**

**^9^ St Francis University College of Health and Allied Sciences, Ifakara, Tanzania**

*, ° equal contribution

**Correspondence to:** Dr. Fiona Vanobberghen, Kreuzstrasse 2, 4124 Allschwil, Switzerland, [fiona.vanoberghen@swisstph.ch](mailto:fiona.vanoberghen@swisstph.ch), +41 (0) 61 284 81 11

**Utangulizi**: Dawa za kupunguza makali ya virusi vya ukimwi (VVU) antiretroviral (ART) kwa wanawake wajawazito na wanaonyonyesha wanaoishi na VVU huwaweka watoto wao ambao hawajaambukizwa na VVU (HEU) katika mazingira ya kupata dawa, jambo ambalo linaweza kuwaathiri. Tulitathmini athari za ART anazotumia mama zenye mchanganyiko wa dolutegravir dhidi ya mchanganyiko wa efavirenz na viashiria vingine vinavyoweza kuathiri matokeo ya ukuaji kwa watoto HEU waliozaliwa na wanawake wanaoishi na VVU vijijini mwa Tanzania.

**Mbinu**: Katika utafiti huu wa uangalizi wakudi la watu wanaou ishi na VVU, tulitumia data kutoka kwa kundi la Uangalizi la VVU linalojulikana kwajina la **Kilombero and Ulanga Antiretroviral Cohort (KIULARCO),** ili kutathmini matokeo ya ukuaji hadi umri wa miezi 18 ya watoto HEU waliozaliwa na wanawake wanaoishi na VVU wanaohudhuria Kliniki ya Magonjwa ya Kudumu ya Ifakara katika maeneo ya vijijini mwa Tanzania kati ya Mei 2012 na Desemba 2024. Tiba ya kwanza ya ART iliyopendekezwa ilibadilika kutoka kutegemea efavirenz hadi kutegemea dolutegravir kuanzia mwaka 2019 na kuendelea. Tulitumia taarifa (data) za ufuatiliaji hadi tarehe 3 Mei 2025 kutoka kwenye mfumo wa kuhifadhi data. Matokeo Msing yalikuwa uwiano (Z-score) wa urefu-kwa-umri na uzito-kwa-urefu, zilizofafanuliwa kwa kutumia viwango vya ukuaji wa watoto vya shirika la afya ulimwenguni WHO, na matokeo ya ziada yalijumuisha ufupi (kudumaa) na uzito mdogo(unyafuzi), vilivyofafanuliwa kama upotovu wa >2 chini ya kiwango cha kawaida cha WHO. Tulipata amatokeo endelevu kwa kutumia njia inayojulikana kwa jina la **l**inear random effects models na matokeo sababu kwa kutumia milinganyo ya makadirio ya jumla ya kihisabati yajulikanayo kwa jina la logistic generalised estimating equations.

**Matokeo**: Kati ya watoto wachanga 1019 waliozaliwa na akina mama 844, jozi 491 (48%) za mama na mtoto zilihusishwa katika kundi la efavirenz na 528 (52%) katika kundi la Dolutegravir. Dawa za ART zilianzishwa kwa wamama 569(56%) kabla ya mimba, Jozi na mamma na mtoto wakati wa mimba ilikua ni wamama 339(33%), na wakati au baada ya kujifungua ili kua ni 111(11%). Wakati wa kujifungua, wamama walikua na wastani wa umri wa miaka 31(wigo wa robo kati ya 26-36) na 742 (78%) ya akina mama walikuwa na maambukizi ya VVU hatua ya WHO ya I/II. Kwa ujumla, watoto 526 (52%) walikuwa wa kiume na 968 (96%) walizaliwa kwa wakati yaani miezi tisa. Watoto walikuwa na mwelekeo wa urefu na uzito ulio chini ikilinganishwa na viwango vya rejeleo vya WHO, bila kujali jinsia au aina ya ART. Katika uchambuzi elezi, kiwango cha urefu mfupi kilichopimwa katika kila ziara katika kipindi cha miezi 18 ya kwanza ya maisha kilikuwa kati ya 18-31% katika kundi la efavirenz na 8-26% katika kundi la dolutegravir, huenda hali hii ikachangiwa na usajili katika miaka ya baadaye ya kalenda katika kundi la dolutegravir. Katika uchambuzi ulioboreshwa, hatukuona tofauti katika matokeo ya ukuaji kulingana na aina ya tiba ya ART.

**Ufasiri:** Katika utafiti huu mkubwa wa kundi, hatukupata uhusiano wowote kati ya aina ya tiba ya ART ambayo wa mama wanao ishi na VVU wanatumia na ukuaji wa watoto wachanga (HEU), lakini ukuaji uliendelea kuwa chini ya viwango vya rejeleo vya WHO. Matokeo yetu yanaunga mkono matumizi endelevu ya dolutegravir kama dawa ya kwanza inayopendekezwa ya ART na yanasisitiza hitaji la utafiti zaidi na uingiliaji kati uliolengwa ili kuboresha afya ya idadi inayoongezeka ya watoto wachanga wasioambukizwa VVU walio zaliwa na wamama wanaoishi na VVU.

**Ufadhili:** Utafiti huu ulifadhiliwa na Taasisi ya Goldschmidt-Jacobson na Taasisi ya Mathieu.
